# Acute compartment syndrome in the hand due to cutaneous anthrax: Case report and literature review

**DOI:** 10.1016/j.idcr.2025.e02395

**Published:** 2025-10-08

**Authors:** Muhammed Kazez, Oğuz Kaya, Yasemi̇n Kırık, Ali̇ Sami̇ Şeker

**Affiliations:** a Department of Orthopedics and Traumatology, Elazig Fethi Sekin City Hospital, Elazig Turkey; b Department of Infectious Diseases and Clinical Microbiology, Elazig Fethi Sekin City Hospital, Elazig, Turkey

**Keywords:** Acute compartment syndrome, Insect bite, Hand, Fasciotomy, Cutaneous anthrax

## Abstract

Cutaneous anthrax, a rare but serious infection caused by the bacterium Bacillus anthracis, generally responds well to medical treatment. However, in some cases, it can lead to serious complications such as meningitis, septic shock, and compartment syndrome. This case report presents a 71-year-old male patient who developed cutaneous anthrax infection following an insect bite, followed by acute compartment syndrome. The patient initially presented with a painless skin lesion, but despite antibiotic treatment, the lesion rapidly deteriorated. The patient was admitted to the inpatient department, started on intravenous antibiotic therapy and closely monitored, but developed acute compartment syndrome in his hand. Emergency fasciotomy was performed to prevent complications of compartment syndrome, and good results were achieved with wound care and skin grafting. This case highlights the importance of early diagnosis and a multidisciplinary approach in the treatment of cutaneous anthrax complicated by compartment syndrome. Timely intervention, including fasciotomy and appropriate wound care, can lead to successful treatment outcomes.

## Highlights

• Cutaneous anthrax may rarely progress to acute compartment syndrome.
• Urgent fasciotomy prevented permanent ischemic damage in the upper limb.
• Early inpatient monitoring enabled timely surgical decision-making.
• Surgical and infectious disease teams collaborated for optimal care.
• This case emphasizes multidisciplinary management in severe infections.

## Introduction

Anthrax disease is caused by the bacterium Bacillus anthracis, which is usually transmitted from animals [1]. There are three different clinical forms of the disease: cutaneous, pulmonary, and gastrointestinal [1]. Cutaneous anthrax usually develops after slaughter, skinning, and, very rarely, insect bites, typically following direct contact [2]. Inhalation of anthrax spores from an infected animal is known to cause pulmonary anthrax, while consumption of infected meat is known to cause gastrointestinal anthrax [2]. Cutaneous anthrax accounts for 95 % of all anthrax cases, and mortality rates range from 10 % to 40 % in untreated cases and less than 1 % in cases receiving appropriate treatment [3]. In most cases, cutaneous anthrax heals with medical treatment and does not lead to complications [4]. However, the clinical course of anthrax can lead to serious and complicated complications in some cases, such as meningitis, septic shock, and compartment syndrome [4]. Compartment syndrome caused by anthrax is a rare but serious complication that can lead to morbidity [5]. In this study, we aimed to analyse a case of compartment syndrome in the hand following cutaneous anthrax, which is very rare but can cause serious morbidity.

## Case presentation

**Fig. 1:**
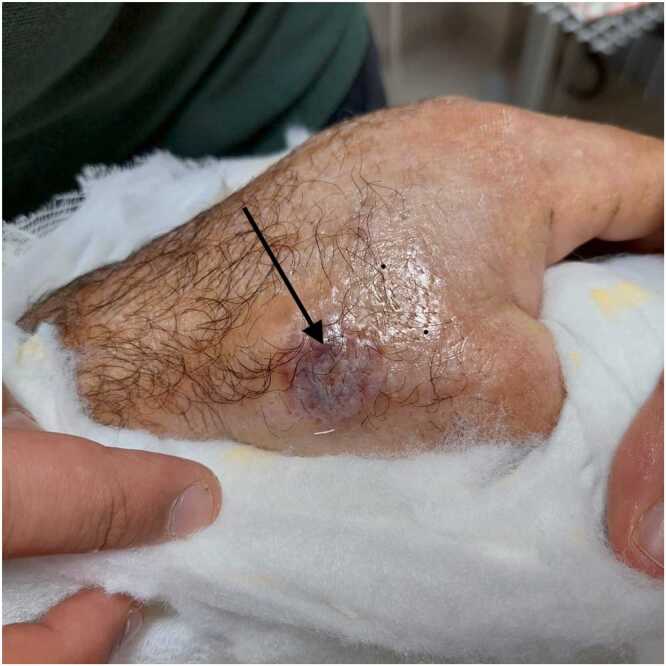
Cutaneous lesion at the level of the 1st metacarpophalangeal joint on the dorsal aspect of the left hand at the time of the patient's presentation to our emergency department / resting splint was applied due to swelling and pain in the patient's hand.

**Fig. 2:**
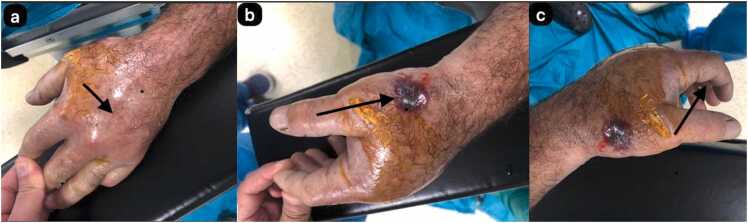
The swelling in the patient's hand increases markedly **(a)**, the lesion enlarges and becomes a haemorrhagic pustule with discharge **(b)**, passive extension of the fingers is very painful **(c)**.

**Fig. 3:**
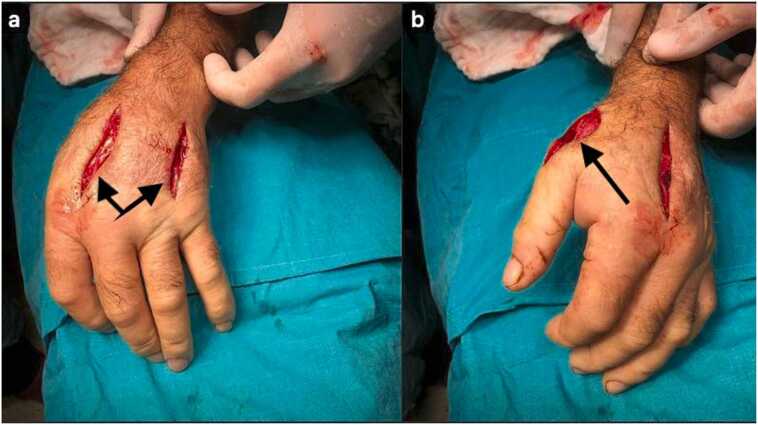
Fasciotomy was performed dorsal to the left hand at the level of the 2nd and 4th metacarpals **(a)**, and the pustular area was excised **(b)**.

**Fig. 4:**
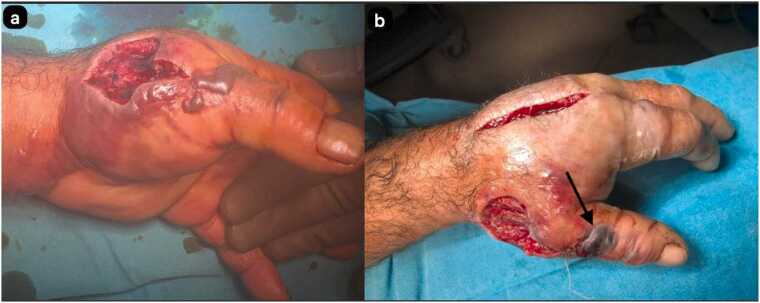
The oedema in the hand has decreased slightly, but it is still markedly oedematous **(a)**, another pustular lesion is evident on the dorsal aspect of the thumb **(b)**.

**Fig. 5:**
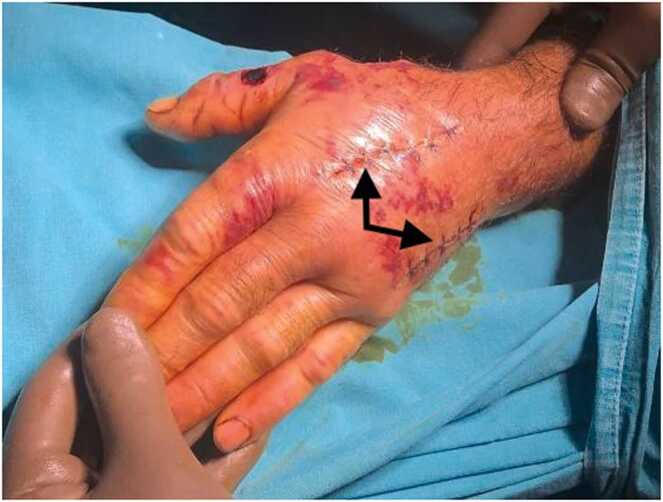
The incisions for fasciotomy were closed primerally on day 10 after the tissue oedema decreased.

**Fig. 6:**
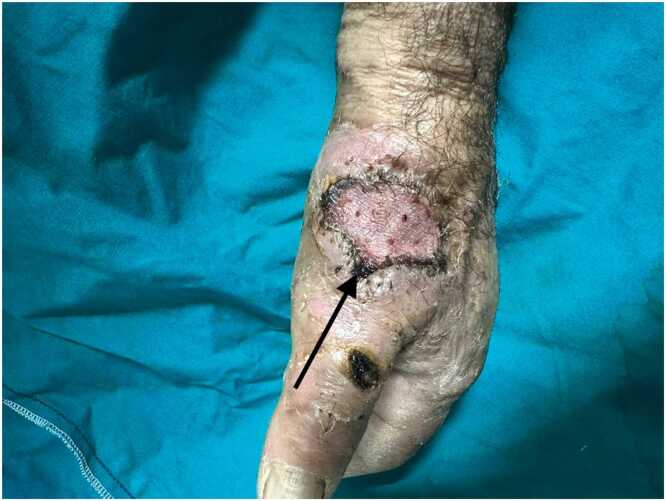
The skin defect was closed with split thickness skin graft on day 13.

**Fig. 7:**
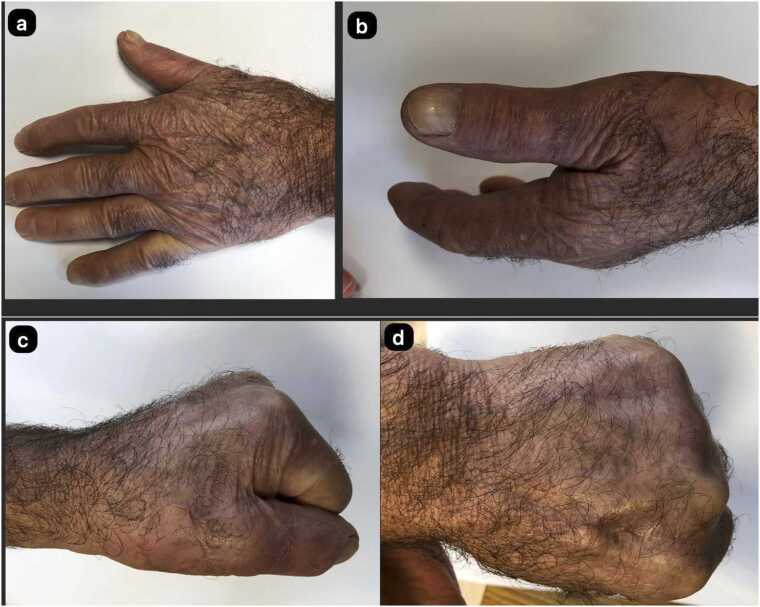
At the 3rd month follow-up of the patient, it was observed that the wounds on the hand healed completely and the hand functions were good **(a-d)**.

The 71-year-old male patient was involved in animal husbandry. He had no known comorbidities other than controlled type 2 diabetes mellitus. He presented to his family physician with a complaint of a painless, itchy skin lesion on his left hand that had started with an insect bite and lasted for a week. Penicillin was prescribed by the general practitioner. Due to the enlargement of the skin lesion on his hand, the patient was referred by his general practitioner to the emergency department of a hospital that was a level 3 trauma centre. Upon evaluation in the emergency department, a minimally painful, 1 × 1 cm oval, erythematous, and pruritic papule-like skin lesion was observed on the dorsal side of the left hand at the level of the first metacarpophalangeal joint (Fig. 1). The patient's temperature was 36.7 °C on admission. The patient could not recall any history of trauma but reported a history of insect bites approximately one week prior. An X-ray was performed to rule out fracture and possible foreign body penetration. No pathology was detected, and the patient was admitted to the orthopaedics and traumatology department for circulation monitoring and further investigation and treatment due to pain and increasing swelling in the hand and fingers. A short arm splint was applied for pain control (Fig. 1) and an infectious diseases consultation was requested for the skin lesion. The patient was evaluated by the infectious diseases department; microscopic evaluation of a Gram-stained preparation revealed Gram-positive bacilli forming a chain-like pattern. The diagnosis of cutaneous anthrax was clinically supported by the occupational exposure history, typical cutaneous lesion morphology, and Gram-positive bacilli forming a chain pattern in the Gram-stained smear; treatment was continued despite negative culture results due to clinical correlation. As PCR/rapid testing facilities were not available at our hospital, the diagnosis was supported by clinical-laboratory correlation and treatment response and treatment with penicillin G (4 million units every 4 h) and ciprofloxacin (400 mg every 8 h) was initiated. Fasciotomy surgery was performed on the patient due to the development of necrosis in the ulcerated area and the onset of compartment syndrome at 48 h of treatment. Laboratory findings: WBC: 14.50 × 10 ³ /µL, sedimentation rate 3 mm/hour, CRP 14.5 mg/dL. The next day, the patient presented with complaints of shivering, a temperature of 39 °C, and significant increase in swelling and pain in the hand (Fig. 2a). The lesion gradually enlarged and took on the appearance of a haemorrhagic pustule associated with superficial erosion of the pustular area and increased peripheral capillary extravasation (Fig. 2b). Bleeding was associated with the edges of the haemorrhagic pustule; no active bleeding was observed from the eschar surface. Passive extension of the wrist and fingers was painful (Fig. 2c). Under general anaesthesia, fasciotomy was performed in all compartments via an incision on the dorsal aspect of the left hand at the 2nd and 4th metacarpal lines (Fig. 3a). The pustular area was excised (Fig. 3b). Samples were taken from the pustular area for microbiological culture and pathological examination, and wound dressings were applied. The patient was followed up in the ward during the postoperative period. Pain complaints decreased, but intermittent fever persisted. After 48 h, wound debridement and dressing change were performed under sedation anaesthesia. A large amount of exudate was observed in the fasciotomy area. The swelling in the hand was observed to have slightly decreased but was still markedly oedematous (Fig. 4a). Another pustular lesion was evident on the dorsal surface of the thumb (Fig. 4b). Biopsy samples taken during the initial surgical procedure were interpreted as Gram-positive bacilli suggestive of Bacillus spp. Cultures taken during the same session were negative. Broad-spectrum antibiotics (carbapenem and linezolid) were initiated for possible sepsis prophylaxis, based on the infectious disease specialist's opinion. The incisions made for fasciotomy were primarily closed after the tissue oedema had subsided (on the 10th day) (Fig. 5). The area with the pustular wound was monitored with a mesh dressing. Antibiotic therapy was continued for 14 days. After the wound area was cleaned and the patient's clinical and laboratory findings improved, the skin defect was closed with a partial-thickness skin graft (on the 13th day) (Fig. 6). The patient was followed up in the outpatient clinic. The graft site was observed to have healed, with good and pain-free hand function (3-month follow-up) (Fig. 7a-d). Timeline of our case

· **Day 0:** Admission, initiation of IV antibiotics.
· ** h: Acute compartment syndrome** with increased pain and oedema → **emergency fasciotomy**.
· ** h post-op: Dressing/debridement** under sedation; oedema partially subsided.
· **Day 10: Primary closure of fasciotomy incisions.**
· **Day 13: Partial-thickness skin graft to the remaining skin defect.**
**· 3 months: Can be summarised as full function and complete wound healing. (Reviewer response 5)**

## Discussion

Anthrax is known as a highly virulent and contagious, quite aggressive infection (zoonosis) that can be fatal. The most life-threatening complications of the disease are meningoencephalitis and septic shock, which have a 100 % mortality rate [5]. In cutaneous anthrax, the most feared complication, which can be life-threatening and is rarely encountered and described in the literature, is compartment syndrome [6]. In our case, we analysed the compartment syndrome that developed suddenly in the hand due to cutaneous anthrax and the treatment process. There are a limited number of case reports in the literature describing compartment syndrome in the extremities due to cutaneous anthrax. Our study and our experience in the treatment process will contribute to this limited information in the literature. Our patient was involved in animal husbandry, and this occupational exposure was decisive in our suspicion of cutaneous anthrax. Occupational contact is a known risk factor in areas where regional animal husbandry is practised. In our case, there was also a history of an insect bite approximately one week prior; this detail is less commonly reported in the existing literature [7] and makes the case unique. It is noteworthy that despite our patient having received oral penicillin treatment prior to admission to our hospital, the lesions did not regress, and compartment syndrome developed suddenly one day after admission to the ward. This is another difference from the current literature [8] and led us to conclude that patients require close monitoring. Insufficient antibiotic effect, which may be related to the dose/duration of treatment administered during the outpatient period, may also have contributed to lesion progression. In cutaneous anthrax, surgical debridement is not recommended in the early stages of the disease due to the increased potential for septicaemia or septic shock [9]. However, as with compartment syndromes of other aetiologies, surgical indication is absolute and urgent in all cases of compartment syndrome [4]. When acute compartment syndrome develops, surgical indication is absolute, and the principle of avoiding early surgery in anthrax is an exception based on limb-saving considerations. In anthrax, this complication can cause serious morbidity in the patient's extremity, endanger their life, and even cause death [3]. In our presented case, the patient had minimal pain at the time of presentation. Thanks to close monitoring of the patient and control of pain and swelling, compartment syndrome was diagnosed without delay and fasciotomy was performed, thus preventing complications of compartment syndrome due to delay. Fasciotomy was performed in patients with cutaneous anthrax considering the morbidities of compartment syndrome, but this may lead to progression of the disease or sepsis. After the fasciotomy site was covered with a dressing, samples were taken from the pustular area for microbiological and pathological examination. The eschar was not manipulated in the early period; samples were taken from the pustular areas and necrotic tissues were removed following demarcation with conservative serial debridements. During follow-up, debridement was not performed for new pustules due to the possibility of lesion spread and the risk of sepsis. In our case, no pustular lesions were observed in the fasciotomy area after surgical procedures, but an increase in lesions was observed around the initial lesion and on the thumb. We believe that the absence of contamination in the fasciotomy area was due to meticulous surgical procedures and measures taken to prevent contamination. In the literature, fasciotomy procedures have been performed on the forearm and hand in most cases of compartment syndrome due to cutaneous anthrax [5], [6]. In this study, fasciotomy was performed only on the dorsal part of the hand using a double incision because there was no stiffness or pain in the forearm and there was pain and swelling in the hand. In the literature and in this study, penicillin therapy and ciprofloxacin were administered in the preoperative period. In cases where compartment syndrome developed and surgical treatment was required, a combination of broad-spectrum beta-lactam and protein synthesis inhibitor was preferred for sepsis prophylaxis and toxin suppression (Referee response 6) [3]. Again, in line with the literature [8], the fasciotomy site in our patient was closed with primary closure after oedema and wound healing, but unlike the literature, the necrotic area where the anthrax pustule formed was closed with a partial-thickness skin graft to accelerate healing and achieve early function.

Although cutaneous anthrax caused by Bacillus anthracis is very rare, it can lead to serious morbidities such as compartment syndrome; therefore, the patient should be closely monitored and evaluated with a multidisciplinary approach. The treatment of compartment syndrome due to cutaneous anthrax carries additional risks such as the growth of anthrax lesions and the development of sepsis. In this case, early intervention following the development of compartment syndrome due to cutaneous anthrax, close monitoring of the wound with serial debridements, and effective collaboration with the infectious diseases department resulted in a favourable outcome for the patient. Early recognition of the disease, characteristic cutaneous lesions and compartment syndrome, and urgent surgical intervention, particularly fasciotomy combined with immediate initiation of antibiotic therapy, are of vital importance in such cases, as this can be a life-saving procedure.

## CRediT authorship contribution statement

**Ali̇ Sami̇ Şeker:** Writing – review & editing, Visualization, Validation, Methodology, Investigation, Data curation, Conceptualization. **Kirik Yasemi̇n:** Writing – review & editing, Visualization, Validation, Methodology, Investigation, Data curation, Conceptualization. **Oğuz Kaya:** Writing – review & editing, Validation, Supervision, Project administration, Methodology, Investigation, Conceptualization. **Muhammed Kazez:** Writing – review & editing, Writing – original draft, Visualization, Validation, Methodology, Investigation, Conceptualization.

## Author contribution

MK drafted the manuscript. YK and AŞŞ were responsible for patient follow-up and data collection. OK served as the corresponding author and supervised the overall project. All authors contributed equally to case planning, surgical decision-making, literature review.

## Consent

Written informed consent was obtained from the patient for publication of this case report and accompanying images. A copy of the written consent is available for review by the Editor-in-Chief of this journal on request.

## Ethical approval

Not applicable. This case report is based on routine clinical care and did not require institutional ethics committee approval.

## Author agreement

• All authors have read and approved the current version of the manuscript.
• The required **Author Statement** (conflicts of interest, funding, consent, author contributions) has been uploaded as a separate file.
• The corresponding author will complete the official Elsevier Publishing Agreement once the article is formally accepted.

## Funding

This research received no external funding.

## Declaration of Competing Interest

The authors declare no conflict of interest.
